# Within-holding prevalence of sheep classical scrapie in Great Britain

**DOI:** 10.1186/1746-6148-5-1

**Published:** 2009-01-08

**Authors:** Ángel Ortiz-Pelaez, Víctor J Del Río Vilas

**Affiliations:** 1 Centre for Epidemiology and Risk Analysis (CERA), Veterinary Laboratories Agency, New Haw, Addlestone, Surrey, KT15 3NB, UK; 2 Food and Farming Group, Department for Environment, Food and Rural Affairs (Defra), Nobel House, 17 Smith Square, London, SW1P 3JR, UK

## Abstract

**Background:**

Data from the Compulsory Scrapie Flocks Scheme (CSFS), part of the compulsory eradication measures for the control of scrapie in the EU, have been used to estimate the within-holding prevalence of classical scrapie in Great Britain (GB). Specifically data from one of the testing routes within the CSFS have been used; the initial cull (IC), whereby two options can be applied: the whole flock cull option by which the entire flock is depopulated, and the genotyping and cull of certain genotypes.

**Results:**

Between April 2005 and September 2007, 25,316 suitable samples, submitted from 411 flocks in 213 scrapie-affected holdings in Great Britain, were tested for scrapie. The predicted within-holding prevalence for the initial cull was 0.65% (95% CI: 0.55–0.75). For the whole cull option was 0.47% (95% CI: 0.32–0.68) and for the genotype and cull or mixed option (both options applied in different flocks of the same holding), the predicted within-holding prevalence was 0.7% (95% CI: 0.6–0.83). There were no significant differences in the within-flock prevalence between countries (England, Scotland and Wales) or between CSFS holdings by the surveillance stream that detected the index case. The number of CSFS flocks on a holding did not affect the overall within-holding prevalence of classical scrapie.

**Conclusion:**

These estimates are important in the discussion of the epidemiological implications of the current EU testing programme of scrapie-affected flocks and to inform epidemiological and mathematical models. Furthermore, these estimates may provide baseline data to assist the design of future surveillance activities and control policies with the aim to increase their efficiency.

## Background

Scrapie is a neurodegenerative disease affecting small ruminants that belongs to the group of diseases known as transmissible spongiform encephalopathies (TSE). Scrapie became a notifiable disease in 1993 in the UK in accordance with EU Council Directive 91/68/EC [1]. Several studies in the nineteen nineties reported the successful experimental transmission of BSE to sheep by both intracerebral and oral routes [2], the presence of BSE infectivity in sheep brain and spleen [3] and the probable exposure of sheep to feed contaminated with the BSE agent [4,5]. As a result, the increasing concern that BSE could be present in the sheep population triggered further epidemiological and pathogenesis studies in accordance with recommendations from expert committees [6,7]. In particular there was a danger that the presence of scrapie could prevent the detection of BSE in sheep. It was BSE rather than scrapie that was viewed as a potential threat to public health. Despite the unknown origin of BSE, and major gaps in scientific understanding of the basic biology of TSEs [8], there have been some attempts to quantify the dimension of the public health threat with the available data [9-12].

In January 2002 and in accordance with EU Regulation 999/2001 as amended [13], large-scale active surveillance was initiated in the UK in two sheep populations: fallen stock and the healthy animals slaughtered for human consumption. Details of these surveys are described elsewhere [14,15].

The Compulsory Scrapie Flocks Scheme (CSFS) was launched in England and Scotland on July 20^th ^2004 and in Wales on November 1^st ^2004 to enforce EU legislation [16]. The scheme introduced compulsory eradication measures in sheep flocks and goat herds in which classical scrapie was confirmed, again as required by European law. Confirmation of classical scrapie in any surveillance route (passive or active) triggers epidemiological investigations leading to the identification of the holding of origin of the case. If the holding of origin is found, one or more flocks may be declared scrapie-affected. These may exist within the same holding (multiple flocks in the same ownership) or on different holdings that are linked epidemiologically. In Great Britain one of two plans of action in affected sheep flocks may apply. The first one is the genotyping and selective cull of Type 3 and 5 genotypes in ewes and non-Type 1 genotypes in rams, with Type 4 genotype ewes being allowed to be slaughtered for human consumption. The five types, as defined in the National Scrapie Plan , establish decreasing levels of resistance to classical scrapie with ARR/ARR or Type 1 being the most resistant and genotypes with alleles VRQ and non-ARR, the Type 5, the least resistant. The second option is the cull of the entire flock without previous genotyping. In both options, a sample of culled sheep over 12 months of age is tested for TSE in the Initial cull, hereinafter referred to as IC. The flock then enters a three-year restriction period during which all fallen stock (FS) over 18 months of age have to be submitted for testing, and re-stocking is only permitted with animals of resistant genotypes. In addition, a sample of all animals over 18 months of age slaughtered for human consumption is TSE-tested every year during the restriction period in the Annual cull (AC).

Past estimates of within-holding prevalence in Great Britain have been derived by different approaches. A postal survey (PS) of farmers conducted in 1998 reported a median within-flock incidence of 0.37 cases per 100 ewes per year (range: 0.05–6.7) calculated as the number of cases that occurred in the previous 12 months divided by the number of breeding ewes [17]. In a follow-up postal survey in 2002, Sivam et al. [18] reported a median incidence of 0.32 cases per 100 ewes per year, with only two farms reporting more than 3 cases per 100 ewes per year. Gubbins [19] used a between-flock transmission model with input parameters such as the within-flock incidence derived from the PS, and reported a plausible range for the within-flock prevalence of infection of 0.8–1.2%. Tongue et al. [20] reported a prevalence of infection of 6.6% (95% CI: 4.4–9.5%) in cull sheep from 14 scrapie-affected flocks in Great Britain. In the Netherlands Schreuder et al [21] conducted two parallel surveys involving face-to-face interviews as well as questionnaires sent by mail to farmers, and reported an incidence rate within infected flocks of 1.27-cases/100 ewes/year.

These estimates present limitations: either they suffer from biases, applicable to the PS and studies derived from them [19], or sample sizes are small and hardly representative [20]. They all refer to classical scrapie since atypical scrapie was either unknown or unaccounted for at the design stages.

Data from the testing of the CSFS holdings provide a larger and more representative picture of the scrapie-affected flock population in GB. The objective of this paper is to report the within-holding prevalence of scrapie infection arising from the analysis of CSFS data. Accurate measures of disease frequency within holdings are essential in order to assess the efficacy of disease control policies, and to inform epidemiological and mathematical models that support policy. Reliable prevalence estimates would provide baseline data to assist the design of future surveillance activities and the discussion of the epidemiological implications of the current EU testing arrangements.

## Results

TSE testing commenced for the FS almost immediately after the launch of the scheme whereas the sampling and testing in the IC began on April 1st 2005. Up to 30 September 2007, 484 flocks from 254 holdings had entered the CSFS with an average of 1.9 flocks per affected holding. During the same period, 25,316 suitable samples, submitted from 411 flocks in 213 holdings, were tested with 1.93 flocks on average per tested holding.

The distribution of holdings in the CSFS and those tested during the study period by country of origin is shown in Table 1. Wales is the main contributor with 109 (43%) CSFS holdings and 100 (47%) tested holdings, followed by England with 101 (39.76%) and 77 (36.15%), respectively. Scotland contributed 44 holdings to the CSFS (17.32%) of which 36 (17%) were tested.

**Table 1:** Distribution of CSFS holdings and those tested in the Initial Cull by country

**Country**	**CSFS holdings**	**Tested (IC) CSFS holdings**
England	101 (39.76%)	77 (36.15%)
Wales	109 (42.91%)	100 (46.95%)
Scotland	44 (17.32%)	36 (16.9%)
Total	254	213

Of the 213 holdings included in the dataset for analysis, the whole flock cull was applied exclusively in 44 (20.65%) holdings; the genotype and cull option in 162 (76%) holdings and only seven holdings (3.3%) had both options implemented in different flocks. Passive surveillance was the most frequent source of the CSFS index case with 159 holdings (74.6%), followed by the fallen stock/dead in transit (DIT) surveillance with 44 holdings (20.6%), and the abattoir survey with ten holdings (4.7%). The average number of samples tested per holding in the IC was 119 (median: 128, range: 1–304). Ninety-four holdings had only one CSFS flock (44.13%), sixty-nine had two flocks in the scheme (32.4%), thirty-two three flocks (15%) and eighteen four or more flocks (8.4%).

**Figure 1:**
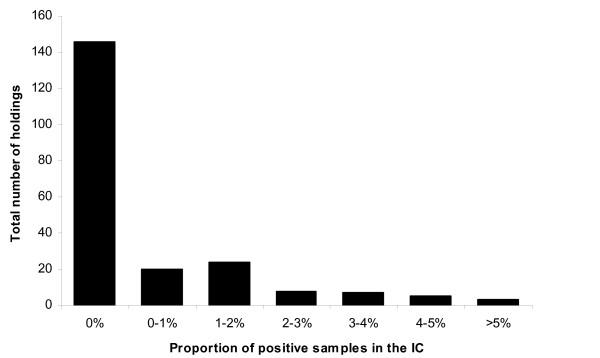
**Frequency distribution of holdings by proportion of positives in the Initial Cull (IC)**.

In 146 (68.5%) of the 213 tested holdings the IC did not detect any case of classical scrapie; twenty (9.4%) had less than one percent of the tested animals positive and the remaining forty-seven holdings (22%) had more than one percent (Figure 1).

The IC testing identified cases of atypical scrapie either in isolation or in combination with classical scrapie within the same holding. Of the 67 holdings confirmed with scrapie, 55 had only classical scrapie (mean number of cases: 2.5, median: 2, range: 1–13), 6 had both classical and atypical scrapie and 6 only atypical cases. Note that only single cases of atypical scrapie were found in the Initial Cull.

The results of the final logistic model are shown in Table 2. None of the variables in the final model appear to be significant at the 0.05 level, although the culling option is of borderline significance (P = 0.059). The overall predicted within-holding prevalence for the whole study population is 0.65% (95% CI: 0.55–0.75). The estimated prevalence by categories of each covariate adjusted for all others is shown in Table 3. For the whole flock cull, the adjusted predicted within-holding prevalence was 0.47% (95% CI: 0.32–0.68). For the second route, the genotype and cull or mixed, the predicted within-holding prevalence was 0.7% (95% CI: 0.6–0.83). Note that the confidence intervals overlap albeit marginally. There are no significant differences in the within-flock prevalence between countries or holdings where CSFS index cases were identified in different surveillance streams. The number of CSFS flocks on a holding does not affect the overall prevalence of scrapie.

**Table 2:** Coefficients of the final multivariate logistic regression model with number of positive cases in a holding as outcome with P values and 95% confidence intervals

**Variable (baseline)**	**Coefficients**	**P value**	**95% CI**	**Odds Ratios**
**Country (England)**				
Wales	-0.12	0.511	-0.5, 0.24	0.88
Scotland	-0.44	0.124	-1, 0.12	0.64
**Number of CSFS flocks within holding**	0.04	0.458	-0.07, 0.15	1.04
Route (WFC*)				
Genotype & cull or mixed	0.39	0.059	0.01, 0.8	1.47
				
**CSFS index case (clinical suspect)**				
Abattoir survey	0.48	0.133	-0.14, 1.12	1.61
Fallen Stock/DIT**	-0.27	0.236	-0.73, 0.18	0.76
**Constant**	-5.25	< 0.001	-5.7, -4.8	

*WFC: Whole flock cull

**DIT: Dead in transit

**Table 3:** Predicted prevalence of the final multivariate logistic regression model for the different categories of the independent variables and 95% confidence intervals

**Variable**	**Prevalence %**	**95% CI**
**Country**		
England	0.68	0.52 – 0.89
Wales	0.69	0.56 – 0.84
Scotland	0.48	0.3 – 0.76
		
**Route**		
WFC*	0.47	0.32 – 0.68
G & C**	0.7	0.6 – 0.83
		
**Source**		
Clinical suspect	0.64	0.54 – 0.77
Abattoir survey	1.12	0.61 – 2
Fallen Stock/DIT***	0.58	0.4 – 0.84

*WFC: Whole flock cull

**G&C: Genotype and cull

***DIT: Dead in transit

## Discussion

The results of this study show that the overall prevalence of scrapie infection within affected holdings is low, with a mean of 0.65%. This estimate is larger than that reported in the postal surveys [17,18], and slightly lower than the plausible range reported by Gubbins [19]. The former referred to prevalence of clinical disease, which is totally dependent on the farmer's diagnostic skills and prone to recall bias and misclassification. The latter was the output of a spatial mathematical model using as input parameters, among others, the results of the PS. In our analysis the prevalence estimate refers to detectable infection by the available approved diagnostic methods at the time of testing. Despite these differences, our results (0.65%) and those of the postal surveys (0.32 per 100 ewes in 2002) are consistent with the predicted ratio of infection to clinical cases (2.2:1) reported by Matthews et al. [22] in a within-flock transmission simulation model.

The interpretation of our results must also take into account the 'a priori' assumptions regarding the tests characteristics and the origin of the CSFS holdings. Not all the CSFS holdings were culled and tested; some farmers, for example, with rare breeds where replacements would be difficult to find, applied successfully for derogation from culling for up to five years. This could have introduced certain selection bias towards larger and common breed flocks and, hence, increase the probability of scrapie occurrence based on these two commonly reported risk factors at the holding level [23]. Furthermore, it was assumed that a perfect test (100% sensitivity) was employed, although for animals in pre-clinical stages of infection this is unrealistic [24]. This could have resulted in the under-estimation of the true within-holding prevalence of infection.

All CSFS holdings, by definition, had to have a previous confirmed case of classical scrapie, the CSFS index case, which was unaccounted for in the analysis in order to be consistent with the objective of the study: to inform on the prevalence of infection. The natural flock dynamics and that of the disease make the prevalence of scrapie variable. Therefore the estimation reported here has to be seen as the proportion of infected cases at the time of the implementation of the eradication measures, usually a few months after the confirmation of the CSFS index case.

Although classical scrapie seems to be present at a low prevalence, several factors may contribute to the observed/apparent low prevalence: an inadequate sample size for the detection of the low within-flock prevalence (see below); the within-flock epidemic is in its early stages resulting in a very low true prevalence and lower negative predictive value of the screening test; the age and genotype distribution leading to individual culling strategies of some affected flocks [20] might have already reduced the prevalence before the index case was confirmed; and the pooling of flocks within the same holding for culling and testing purposes may have generated a lower combined prevalence in the cull population. Other exogenous factors unaccounted for could explain the presence of only the CSFS index case, e.g. if farmers seek to benefit from the favourable regulatory context and compensation schemes linked to the CSFS. This might increase farmers' efforts to find disease. Note here that 75% of the CSFS holdings had the notification of clinical cases as the source of their index cases. This would be in line with previous findings [25] that reported that over 80% of the variability in the incidence of scrapie in the US was the result of reporting artefacts.

The detection of holdings with cases of scrapie in the IC decreased with time as eradication measures were applied. During the first eighteen months of the study period, up to September 2006, 23.3% of the tested holdings had positive cases compared to the 12.8% of the tested holdings in the last twelve months. In the first nine months of 2007 none of the 16 tested holdings presented positive cases. The detection of heavily infected holdings by the regular surveillance sources at the early stages of the CSFS compared to holdings with few or only one infected/diseased animal, the index case, later on, explain this decay in the number of CSFS holdings with further positive cases detected during the testing programme. This apparent decreasing trend in the within-holding prevalence, if true for all scrapie-affected holdings in the population, may have two effects. Firstly a reduction in the detection of new scrapie-affected holdings by the regular surveillance activities, as the probability of detection depends, among other factors, on the within holding prevalence. And secondly, the sampling schemes within scrapie-affected holdings across the EU will no longer have enough power to detect cases unless they adapt regularly as a result of analyses like the one presented here.

The results indicate that the difference in the estimated prevalence between eradication routes was not statistically significant. This was unexpected under the hypothesis of different genotype profiles for the two culling options. However there are no data on genotype profiles of the holdings under the whole flock cull option to confirm this assumption. The estimation of the scrapie prevalence in groups of CSFS holdings culled under the genotype and cull option and with different genotype profiles is an ongoing extension of this study that will allow the confirmation of the impact of the genotype profile at flock level on the ability of the tests to detect infected animals. The selection of animals for testing could have also introduced bias in the cull population in either cull option. For example if cull animals were not randomly selected as required and, as a result, some characteristic associated with scrapie was introduced in the sample, e.g. animals easier to catch as a result of poorer condition.

The current sampling frame set by the EU Regulation 999/2001, calculated to be 95% certain of including at least one positive if the disease is present at a minimum prevalence of 2%, may have also contributed to the observed low prevalence estimates. With a prevalence of 0.65%, the probability of detecting a positive case in the IC drops to 62%. The practical consequences of this reduced ability to detect cases of scrapie are immediate. The targeted testing programme is intended to "gather epidemiological information" [26]. If no further cases are detected in the testing routes of the scrapie-affected flocks, this aim and the likelihood of detecting positive "bought-in" cases to enable trace-back to other scrapie-affected flocks both appear to be compromised. In a wider EU context, the heterogeneity of scrapie prevalence across member states (MS) [27,28] could result in the inadequate testing of the affected flocks, with consequences in the quality of the epidemiological information gathered. It is advisable to conduct an analysis of similar data across MS to ascertain the levels of scrapie in affected holdings and to evaluate the efficacy of the testing programme at the country level. These results also suggest that proposals to relax statutory controls as provided in EU Regulation 727/2007 [29] and subsequently EU Regulation 1428/2007 [30], shortening the period of restriction from three to two years may present a greater risk while, potentially, infected animals remain undetected in the holding.

The number of reported and confirmed cases recorded through passive surveillance is at their lowest levels since scrapie became notifiable in 1993 in GB [31]. This may lead to the perception that scrapie is no longer a problem for the national sheep flock. The impact of the eradication measures on the overall decrease in the incidence of scrapie in GB has to be assessed in the context of the national efforts to eradicate the disease. In this regard, the National Scrapie Plan (NSP) schemes, established to increase the frequency of resistant alleles to classical scrapie in the general population [32], and the individual efforts of a number of farmers who have pursued a similar objective privately, are contributing factors, together with the CSFS, to the low figures of confirmed cases as shown by the core indicator 2.1c "incidence of scrapie" of the Animal Health and Welfare Strategy Indicators of Defra . However it is worth mentioning that both breeding for resistance and the compulsory eradication measures pursue similar objective using very different approaches and timescales. While breeding for resistance reduces progressively the prevalence of susceptible individuals without directly intervening by detecting and removing infected animals, the CSFS causes a real removal of infected/diseased animals from the national flock with the consequent reduction of environmental contamination and reduction of the risk of transmission within and between holdings. If the minimum population of holdings infected with scrapie in mainland GB was 642 in 2004 [33], the actions taken on 213 scrapie-affected holdings under the CSFS and on 193 scrapie-affected holdings under the previous scheme, the Voluntary Scrapie Flocks Scheme (VSFS) [31], have resulted, at the most, in the reduction of almost two thirds of the attributable scrapie risk between 2004 and 2007, assuming that scrapie is in fact eliminated within holding by the myriad of actions under the two schemes (see limitations regarding power of detection above). In the general population, the culling of rams and ewes of susceptible genotypes under the different NSP schemes as per EU legislation [34] may also have cleared infected animals from such flocks. It is however difficult to quantify that risk reduction.

In agreement with Luhken et al. [35] on the detection of atypical scrapie and Benestad et al. [36] on the co-infection of atypical and classical scrapie in the same flock as a rare event, our results show the coexistence of classical and atypical scrapie within the same holding in 9% of the holdings with cases in the Initial cull, where single cases of atypical scrapie were detected. So far there has been only one holding in the CSFS where two cases of atypical scrapie have been confirmed, but from different surveillance routes: the Initial cull and the Fallen Stock. Although speculative, the coexistence of classical and atypical scrapie in a number of holdings raises questions on whether it occurred by chance or there was a link between the occurrence of the two types of scrapie (i.e. by exposure to a similar environmental source). Note that our results only provide a partial image of the frequency of coexistent types of scrapie within the same holding due to the restricted nature of the CSFS in Great Britain where only classical scrapie triggers the inclusion of holdings in the scheme. It is known to the authors that other EU countries have extended the TSE testing to all holding regardless of the type of scrapie of the index case. Specific controls for flocks with atypical scrapie were provided in EU Regulation 727/2007 [29]. Despite the possible spontaneous occurrence of atypical scrapie [36,37] and as a possible collateral effect of the eradication of classical scrapie, the cull of susceptible genotypes to classical scrapie may well increase the prevalence of atypical scrapie relative to classical, if present at all, in CSFS holdings or in those where breeding for resistance has been implemented, by means of an increase in the susceptible population. For the first time, in 2006 there were more confirmed cases of atypical scrapie than classical in the active surveillance in Great Britain . However the increase of susceptible genotypes to atypical scrapie and the increase in the number of atypical cases confirmed in CSFS holdings, due to the statutory Initial Cull and the re-stocking with Type 1 and 2 sheep, do not necessarily imply a significant increase of the overall prevalence of atypical scrapie in the national flock.

## Conclusion

The results of this study have reported the estimated prevalence of classical scrapie in affected holdings using actual data from the Compulsory Scrapie Flocks Scheme (CSFS) in GB. The overall predicted within-holding prevalence was 0.65% (95% CI: 0.55–0.75), consistent with previously reported estimates of prevalence of clinical disease in the postal surveys and predicted ratio of infection to clinical cases. There are no significant differences in the within-flock prevalence between countries and holdings where CSFS index cases were identified in different surveillance streams. The number of CSFS flocks on a holding does not affect the overall prevalence of scrapie. Although the analysis was done using data only from the IC, the results provide a robust estimation of the prevalence of scrapie at the time of implementation of this testing route. The low prevalence reported highlights the uncertainties underpinning the actual impact of the eradication measures in the decline of scrapie incidence and the sampling strategy as per EU legislation, but may, together with analyses of between country heterogeneity, facilitate a review of the legislative objectives for the future.

## Methods

### Data

Test results from the IC, number of animals tested and number confirmed positive, were collated for all holdings where CSFS eradication measures were applied since its launch up to 30 September 2007. Additional covariates were extracted from the CSFS database, including: country of origin (England, Scotland, Wales), culling option (whole flock cull, genotype and cull/mixed), number of CSFS flocks in the holding and the surveillance source (clinical suspect, fallen stock, abattoir survey) of the case that triggered the CSFS declaration, the CSFS index case.

During the IC, a sample of sheep is selected for TSE testing in accordance with the sampling frame established in EU Regulation 999/2001 [13], whereby the sample size is calculated "to be 95% certain of including at least one positive if the disease is present at a minimum prevalence of 2% in the test population". The maximum number of samples, 150, is taken when the eligible cull population within the holding is 500 or more sheep. All animals are sampled and tested if the eligible cull population is 70 or less. [13].

Animals for testing must be randomly selected from the eligible cull population. If there is more than one CSFS flock from the same holding, the calculation of the number of animals to be tested is based upon the pooled number of animals over 12 months of age from all flocks. For holdings with multiple CSFS flocks the estimate of prevalence is then the pooled prevalence of the affected flocks within the holding. Although the declaration of CSFS is at flock level, the estimation of the prevalence will be referred hereinafter as within-holding prevalence, which describes more accurately the main outcome of the analysis.

Brain stem and cerebellum are taken from selected animals for testing. The Bio-Rad TeSeE ELISA is used as screening test in all samples. If positive, Immunohistochemistry (IHC) and VLA Hybrid Western Blot are used in obex and/or cerebellum to confirm the case and to discriminate between classical and atypical scrapie [38].

### Analytical methods

Descriptive analysis of CSFS holdings takes into account country of origin, culling option and the source of the CSFS index case. Tests results from the IC include the number of tested animals per holding and description of positive results by scrapie type, culling option and country of origin.

A multivariate logistic regression model for grouped data with maximum-likelihood estimators and a logit function was used to model the outcome, the number of positive cases in a holding. The binomial distribution was selected in order to account for the increasing probability of detecting a positive case with the increasing number of animals tested. Four covariates (country, surveillance source, number of flocks and culling option) were introduced in the model and tested for interaction. Stata 10 (Stata Corporation Ltd.) was used for the analyses. The estimation of the prevalence was only calculated for classical scrapie. Atypical cases were also confirmed in some flocks and their occurrence is also reported.

## Authors' contributions

AOP designed the study, conducted the analysis and wrote the manuscript. VJDRV critically revised the manuscript. Both authors read and approved the final manuscript.
